# Systematic evaluation of cerebral injury stimulating by underwater infrasound

**DOI:** 10.3389/fphys.2026.1803881

**Published:** 2026-05-13

**Authors:** Xia Liu, Tao Shan, Li Wang, Yuewen Ding, Guolin Cui, Xi Kang, Zeyu Si, Zhennan Wang, Yaozong Pan, Ci Li, Chunying Shi, Yiqun Fang

**Affiliations:** 1 Naval Medical Center, Naval Medical University, Shanghai, China; 2 Department of Human Anatomy, Histology and Embryology, School of Basic Medicine, Qingdao University, Qingdao, China; 3 Qingdao Branch, Institute of Acoustics, Chinese Academy of Sciences, Qingdao, China; 4 Faculty of Information Science and Engineering, Ocean University of China, Qingdao, China; 5 College of Artificial Intelligence and Big Data, Qingdao Institute of Technology, Qingdao, China; 6 School of Physical Sciences, University of Chinese Academy of Sciences, Beijing, China

**Keywords:** underwater infrasound, cerebral injury, subarachnoid hemorrhage, morphological injury, apoptosis

## Abstract

**Introduction:**

Infrasound (<20 Hz) was an inaudible mechanical vibration that threatened public health, especially the central nervous system (CNS) in air environments. In fact, infrasound was also produced by large ships or underwater apparatus, which had potential hazard to underwater operators or divers. However, the propagation characteristics of sound waves in air differed from that in water, and there was no experimental evidence on the potential bioeffects of underwater infrasound. In the present study, we systematically evaluated cerebral injury in Bama mini pigs induced by underwater infrasound.

**Methods:**

Forty-two Bama mini pigs (weighing 10-15 kg) were randomly divided into seven groups. After wearing a respiratory mask, the animals were submerged to a depth of 10 meters and exposed to infrasound at frequencies of 8 Hz (145.4 ± 0.2 dB re 1 μPa, 156.2 ± 1.0 dB re 1 μPa), 12 Hz (145.3 ± 0.2 dB re 1 μPa, 161.9 ± 2.4 dB re 1 μPa), and 20 Hz (145.5 ± 0.3 dB re 1 μPa, 165.9 ± 0.3 dB re 1 μPa). Then cerebral function was assessed using electroencephalography (EEG); morphological injuries were evaluated by HE staining, Nissl staining, and immunostaining; oxidative stress and inflammatory cytokines were detected via ELISA assays; and the underlying molecular mechanisms were explored through RNA sequencing and transcriptome analysis.

**Results and discussion:**

Compared with the control group, underwater infrasound at 8 Hz led to alterations in EEG in partial brain regions, but with no significant changes in the 12 Hz and 20 Hz groups. In contrast, underwater infrasound at 20 Hz and 12 Hz directly induced histological injury, decreased neuron density, and increased cell apoptosis immediately, but had no impact on histological and neuronal injury induced by 8 Hz exposure. Additionally, there was no significant influence on oxidative stress among all groups. Finally, transcriptome analysis revealed activation of immune and cell apoptosis pathways and key genes. These results provided direct evidence for the adverse effects of underwater infrasound on brains.

## Introduction

Infrasound generally refers to sound waves with frequencies between 10^−4^ and 20 Hz, which are imperceptible and inaudible to humans. It can be produced by natural sources, such as ocean waves and earthquakes and can also be generated by artificial sources, such as industrial installations, vibration of mechanical equipment inside enclosed spaces, wind turbines, and transportation (Ma et al., 2015). Infrasound is characterized by strong vibration, high penetration, low attenuation during long-distance propagation, and difficulty in shielding (Zhang et al., 2023).

Currently, it is known that infrasound is harmful to different organs and tissues (Lousinha et al., 2018; Xia et al., 2025). Among these organs and tissues, the central nervous system (CNS) is the most vulnerable, especially the brain. In previous studies of *in vitro* cultured hippocampal neurons, infrasound at a frequency of 16 Hz and a pressure level of 130 dB re 20 μPa for 1 h caused axonal degeneration by increasing Ca2^+^ influx (Cheng et al., 2012); in addition to neurons, infrasound also induced significant astrocytic and microglial activation in hippocampal regions, and these activated glial cells released proinflammatory cytokines, tumor necrosis factor alpha (TNF-α) and interleukin-1β (IL-1β), consequently leading to further neuronal impairment (Shi et al., 2013). Consistent with these *in vitro* experiments, rats exposed to 16 Hz, 130 dB re 20 μPa or 8 Hz, 140 dB re 20 μPa infrasound showed direct hippocampal damage with increased cell apoptosis, as well as impaired learning and memory abilities (Jiang et al., 2022). In humans, because the frequencies of infrasound are lower than human hearing threshold, it is inaudible to the human ear but has a detrimental impact on the brain (Salt and Hullar, 2010). It was reported that some aircraft technicians who work daily in an infrasonic environment showed abnormal brain magnetic resonance imaging patterns or a fairly high incidence of late-onset epilepsy (Castelo Branco and Rodriguez, 1999). Moreover, other CNS symptoms, such as dizziness, severe vertigo, unique and sudden episodes of non-convulsive neurological deficit and delayed multimodal evoked potentials, were also frequently observed in these individuals (Branco and Alves-Pereira, 2004; Cai et al., 2014). These results indicated that infrasound might directly disturb the cerebral function.

With the increasingly widespread use of large ships and underwater equipment, infrasound generated by these vessels and systems has become another important source of infrasound, which poses a potential health hazard to underwater operators or divers. However, the propagation characteristics of sound waves in air are different from those in water (Dong et al., 2023). The acoustic impedance of air is approximately 415 MKS rayls (kg/m^2^s), whereas the acoustic impedance of water is approximately 1.5 × 10^6^ MKS rayls, which is comparable to the average acoustic impedance of the human body (1.6 × 10^6^ MKS rayls). The impedance mismatch directly causes most airborne sound reaching the human body to be reflected, whereas sound in water passes directly into the body (Pestorius et al., 2010). In addition, sound waves can also induce water fluctuations, which further act on the organism through a “water hammer” effect. This effect might also differ from the bioeffects of infrasound in air.

Currently, whether underwater infrasound can cause systemic cerebral injury remains unclear. Because the brain morphological structure of porcine is similar to that of humans, we used porcine for further systemic evaluation of cerebral injury induced by underwater infrasound exposure in the present study. As human organs have their own inherent vibration frequencies (e.g., the head, 8 Hz–12 Hz; the thoracic cavity, 4 Hz–6 Hz; the heart, 5 Hz; the abdominal cavity, 6 Hz–9 Hz), frequencies of 8 Hz, 12 Hz, and 20 Hz at average sound pressure levels (SPLs) of 145 dB re 1 μPa and 160 dB re 1 μPa (all dB values mentioned below are referenced to 1 μPa) were used as infrasound stimuli in the present study. Then, the instantaneous functional status of the brain was evaluated by electroencephalogram; morphological damages was assessed by HE staining, Nissl staining, and immunostaining; finally, the underlying molecular mechanisms were explored by RNA sequencing and transcriptome analysis.

## Materials and methods

### Animals and experimental procedures

All experimental procedures were carried out in accordance with local guidelines for the ethical use of animals and the National Institutes of Health’s Guidelines for the Care and Use of Laboratory Animals (NIH publication 23–80, revised in 2011), and all protocols were approved by the Animal Care and Use Committee of Qingdao University (Approval No. QDU-AEC-2024641). BAMA mini pigs (weight: 10 kg to 15 kg) were purchased from Jinan Xijiaoling Breeding Center (Lindholm and Lundgren, 2009).

Briefly, the animals were anesthetized by intraperitoneal injection of 50 mg/kg sodium pentobarbital, and electroencephalogram was then performed to evaluate cerebral function. After being fitted with a respiratory mask, the animals were placed underwater at a depth of 10 meter, which is widely used for engineering construction and maintenance, emergency rescue, aquatic product fishing, scientific research and exploration, and recreational diving.

**Figure 1:**
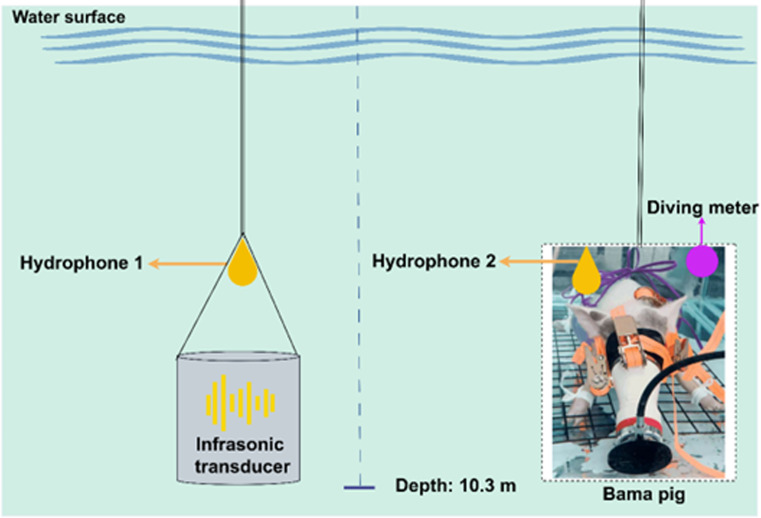
The schematic overview of the experiment (Image created with Figdraw.com).

The infrasonic transducer was designed and produced by the Qingdao Branch of the Institute of Acoustics, Chinese Academy of Sciences. Its weight was about 150 kg and it could emit a continuous infrasonic wave at 8 Hz–20 Hz. Based on previous studies of inherent human vibration frequencies (8 Hz–20 Hz) and frequency selection in air infrasound animal experiments, frequencies of 8 Hz, 12 Hz, and 20 Hz were used for underwater infrasound exposure, with average sound pressure levels (SPLs) of 145 dB and 160 dB. In previous studies, it was reported that when the average sound pressure level of infrasound was set at 140 dB–172 dB, human individuals could endure exposure for 2 min (Gužas and Viršilas, 2009). As a result, the duration of underwater infrasound exposure was set at 2 min. A control group was also established, in which the animals were placed underwater at 10 meter for 2 min without infrasound exposure. During the entire experimental process, a diving meter was placed on the animal life support system platform, as shown in **Supplementary Figure 1**. It recorded the depth and duration at maximum depth, from which the pressure was calculated. In addition, two hydrophones were used for SPL calibration. Briefly, at the vertical position of the underwater acoustic transducer, hydrophone 1 was used to measure the acoustic energy parameters emitted by the transducer; at the horizontal position of the Bama pig, hydrophone 2 was used to measure the acoustic energy parameters received by the animal. The schematic overview of the experiment is shown in **Figure 1**, and the physical layout diagram is presented in **Supplementary Figures 1A, B**.

### Electroencephalogram

The Bama pigs were anesthetized using the aforementioned method. Electroencephalography (EEG) recordings were performed before the animals entered the water and after they emerged. Following anesthesia, electrodes were placed according to previously established methods for porcine EEG recording (Cymbalyuk et al., 2024) as follows: T3 and T4 electrodes were positioned 2 cm posterior to the left and right orbital margins, respectively, corresponding to the temporal regions; P3 and P4 electrodes were placed at the one-third points along the line connecting T3 and T4 across the cranial vertex, corresponding to the parietal regions; C3 (C4) electrodes were placed 5 cm anterior to P3 (P4), and O1 (O2) electrodes were placed 5 cm posterior to P3 (P4). The C3 (C4) electrodes represented the left (right) frontal regions, while the O1 (O2) electrodes represented the left (right) occipital regions. Odd-numbered electrodes were positioned on the left side of the sagittal suture, and even-numbered electrodes were positioned on the right side. Reference electrodes were placed on the left and right ear margins of the pigs. This setup monitored the eight major functional areas of the pig brain: the left and right frontal, parietal, occipital, and temporal lobes (**Supplementary Figure 1C**).

After obtaining the EEG monitoring data, standardized statistical analysis of energy value was performed on brainwaves from each region as follows: the recorded EEG waveforms were exported as EDF files and processed using Anywave software for power spectral density (PSD) analysis to obtain raw energy data (Nuwer, 1988). The data were then imported into MATLAB to generate comparative graphs of pre- and post-immersion curves. For each lead, the first 30 raw values from the PSD analysis were selected, and their average was used as the baseline. Each raw value was divided by the baseline to obtain a calibrated value (δ wave: 1 Hz–3 Hz; θ wave: 4 Hz–7 Hz; α wave: 8 Hz–13 Hz; β wave: >14 Hz). The average calibrated value for each waveband was calculated. GraphPad Prism 8 was used for intragroup and intergroup statistical analysis.

### Paraffin section preparation

After the animals were sacrificed, the brain was perfused with physiological saline. Subsequently, tissue samples from different brain regions were collected. To ensure consistent anatomical localization across animals, tissues from the frontal, temporal, parietal, and occipital cortices of the right hemisphere were obtained via coronal sectioning, with a size of 1 cm × 1 cm × 0.3 cm. All samples were collected from corresponding anatomical landmarks based on a porcine brain atlas to maintain inter-animal consistency. These tissues were fixed in 4% paraformaldehyde, then placed in 4% buffered paraformaldehyde for 72 h, and sectioned into 5-μm-thick slices. Because the established roles of the frontal and temporal cortices include cognitive function, memory processing, and auditory integration, two sections per frontal cortex and temporal cortex per animal were selected; the parietal and occipital cortices were each sampled with one section per animal for subsequent hematoxylin–eosin (H&E) staining, Nissl staining, and immunostaining.

### Histological staining and Nissl staining

After dewaxing with xylene and washing with various concentrations of ethanol, the slices were stained with hematoxylin for 15 min using the HE reagent kit (Solarbio, G1120). After 30 s of differentiation, the slices were placed in eosin solution for 2 min. After rinsing with tap water, the slices were dehydrated, rendered transparent, and sealed, and their morphology was observed under a microscope (Nikon Ni-U, Tokyo, Japan). The slices were also stained with a Nissl staining kit (LEAGENE, DK0022, Huaibei, Anhui Province, China). The slices were submerged in cresyl violet solution and incubated at 56 °C for 1 h. They were then differentiated using Nissl differentiation reagent for 2 min. The sections were then examined for the count, morphology, and distribution of Nissl bodies. Finally, ImageJ software was used to quantify the Nissl body density per area.

### Assessment of subarachnoid hemorrhage severity

To evaluate the severity of subarachnoid hemorrhage (SAH) induced by underwater infrasound exposure, a semi-quantitative scoring system was employed. After the animals were sacrificed, the brains were carefully removed, and the ventral surface was examined for the presence and distribution of subarachnoid blood. The scoring method was adapted from previously established protocols for SAH grading in experimental models (18DDI). Briefly, the basal cistern was divided into six regions: (1) left anterior cerebral artery territory, (2) right anterior cerebral artery territory, (3) left middle cerebral artery territory, (4) right middle cerebral artery territory, (5) anterior communicating artery complex, and (6) basilar artery territory. Each region was scored on a scale of 0 to 3 based on the amount of visible subarachnoid blood: 0, no blood; 1, minimal blood with visible vessels; 2, moderate blood with vessel partially obscured; and 3, severe blood with vessels completely obscured by clots. The scores from all six regions were summed to obtain a total SAH score (range 0–18). The scoring was performed by two independent investigators blinded to the experimental groups, and the average score was used for statistical analysis.

### Immunofluorescence staining

To evaluate the brain recovery after cerebral ischemia in rats, immunofluorescence staining was performed. Brain slices were washed with PBS, incubated with 20% FBS, and then incubated with primary antibodies at 4 °C overnight. Anti-NeuN antibody (rabbit, 1:100, ABclonal, Wuhan, Hubei, China, Cat# A0951, RRID: AB_2757475) and anti-Tuj-1 antibody (rabbit, 1:300, Abcam, Cambridge, UK, Cat# ab18207, RRID: AB_444319) were used to evaluate neuronal survival. Then, the corresponding goat anti-rabbit IgG H&L (Alexa Fluor^®^ 594) (1:500, Abcam, Cat# ab150080, RRID: AB_2650602) and goat anti-mouse IgG H&L (Alexa Fluor^®^ 488) (1:500, Abcam, Cat# ab150113, RRID: AB_2576208) secondary antibodies were added to the slices for 1 h at 25 °C. Finally, 4′,6-diamidino-2-phenylindole (ab104139, Abcam) was used to stain nuclei on the glass slides. Because CI/R typically induces cell apoptosis (Liu et al., 2023), the TdT-mediated dUTP nick end labeling (TUNEL) assay kit (40307ES20, YEASEN, Shanghai, China) was used to assess cell apoptosis. To ensure consistent anatomical localization across animals, coronal sections were prepared from the frontal, temporal, parietal, and occipital cortices. The frontal cortex (including the prefrontal region) and temporal cortex were sampled at a higher frequency (two sections per region per animal) because of their established roles in cognitive function, memory processing, and auditory integration, which are relevant to the infrasound exposure paradigm. The parietal and occipital cortices were each sampled with one section per animal. All sections were collected from corresponding anatomical landmarks based on a porcine brain atlas to maintain inter-animal consistency. Six slices were randomly selected from each group, and apoptotic cells were counted using ImageJ.

### Detection of oxidative stress and inflammatory cytokines

Following water exit, the animals first underwent EEG, ECG, echocardiography, and venous blood collection. Subsequently, they were euthanized for tissue sample collection from various sites. The entire process was completed approximately 2 h after infrasound exposure. To investigate the impact of acoustic stimulation on oxidative stress and inflammatory cytokines, we prepared frontal brain tissue protein extracts for the measurement of oxidative stress indicators, including glutathione (GSH) (Catalog#G4305-96T, Servicebio) and total superoxide dismutase (T-SOD (Catalog#G4306-96T, Servicebio), as well as quantitative analysis of tumor necrosis factor-α (TNF-α) (Catalog#JM-01217P1, JINGMEI BIOTECHNOLOGY) and Interleukin-6 (IL-6) (Catalog#JM-01252P1, JINGMEI BIOTECHNOLOGY) using ELISA kits. All experimental procedures adhered strictly to the manufacturer’s protocols.

### Transcriptome sequencing

Transcriptome sequencing of the control group, 8 Hz/145.4 ± 0.2 dB group, 8 Hz/156.2 ± 1.0 dB group, 12 Hz/145.3 ± 0.2 dB group, 12 Hz/161.9 ± 2.4 dB group, 20 Hz/145.5 ± 0.3 dB group, and 20 Hz/165.9 ± 1.3 dB group (n = 3) was performed to clarify the effects of underwater infrasound on differentially expressed genes in the brains of Bama pigs. Frontal cortex samples from injured brain were collected and sequenced by Sangon Biotech. Finally, figures of the differentially expressed genes were generated using bioinformatics analysis.

### Statistical analysis

All results were expressed as the mean ± standard deviation. Statistical analysis was performed using GraphPad Prism 8.0.1 (GraphPad Software, La Jolla, CA, USA, www.graphpad.com). To compare data between two groups, Student’s t-test was used. For comparison among multiple groups, two-way analysis of variance followed by Tukey’s *post hoc* test was used. A P-value <0.05 was considered statistically significant.

## Results

### Alteration of cerebral electrical activity by underwater infrasound

**Figure 2:**
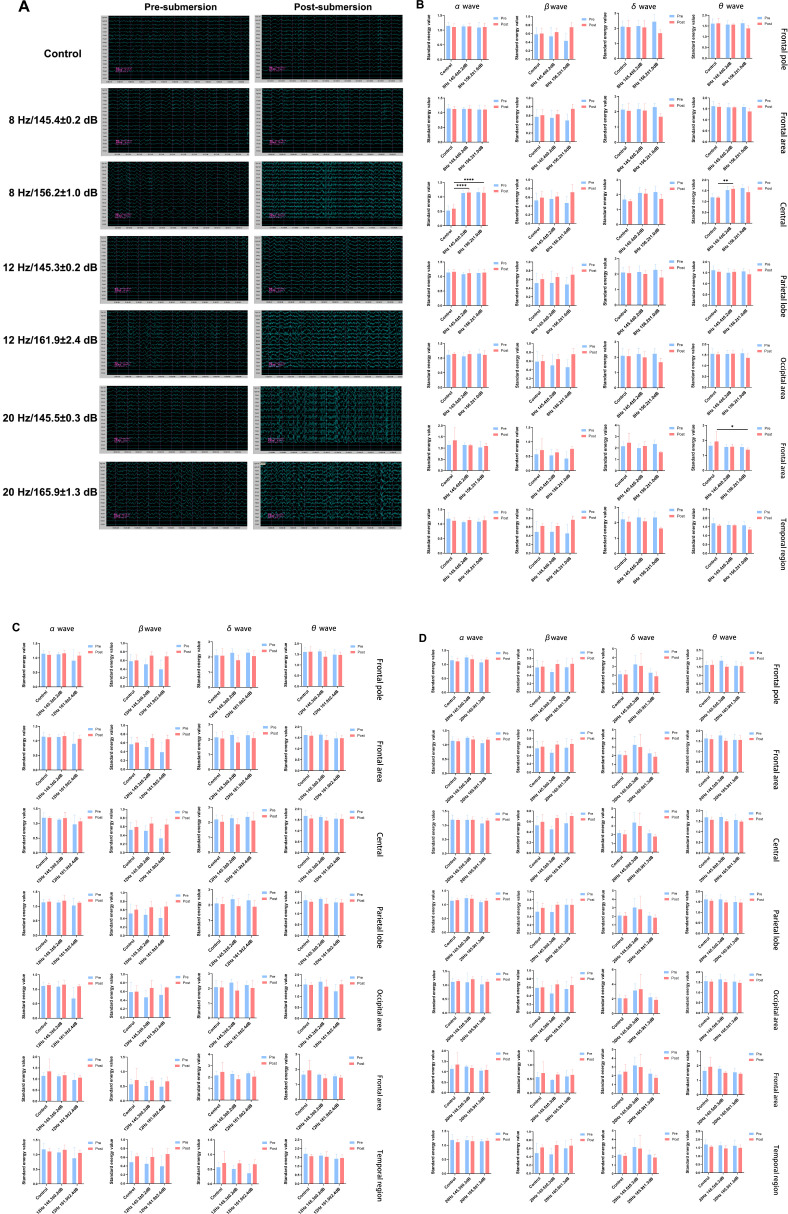
The EEG waveform and standardized energy value statistical analysis of various infrasound exposure. **(A)** The EEG waveform of various infrasound exposure; **(B)** The standardized energy value statistical analysis of EGG in 8 Hz (145.4 ± 0.2 dB, 156.2 ± 1.0 dB) groups; **(C)** The standardized energy value statistical analysis of EGG in 12 Hz (145.3 ± 0.2 dB, 161.9 ± 2.4 dB) groups; **(D)** The standardized energy value statistical analysis of EGG in 20 Hz (145.5 ± 0.3 dB, 165.9 ± 1.3 dB) groups. n = 6. Data represent means ± SD. *P <0.05, **P <0.01, ****P < 0.0001.

Electroencephalography (EEG) was used to examine brain activity abnormalities. As shown in **Figure 2A**, there was no significant difference between pre-submersion and post-submersion in the control group. However, after infrasound exposure, alterations in EEG waveforms were observed in various infrasound exposure groups, including abundant paroxysmal abnormal discharges on EEG, such as spike waves and multiple spike waves. These abnormal EEG discharges are labeled in a zoomed-in view in **Supplementary Figure 2**. To further characterize the specific EEG alterations induced by infrasound exposure, standardized statistical analysis of energy was performed. As shown in **Figure 2B**, compared with the control group, there were significant alterations in the standardized energy values of the α and θ waves in the central region of the 8 Hz/145.4 ± 0.2 dB group; there were also significant alternations in the standardized energy values of the α wave in the central region and the θ wave in the frontal region of the 8 Hz/156.2 ± 1.0 dB group. However, in various cerebral regions, no significant alternations were observed in the standardized energy values in the 12 Hz/145.3 ± 0.2 dB group, 12 Hz/161.9 ± 2.4 dB group, 20 Hz/145.5 ± 0.3 dB group, and 20 Hz/165.9 ± 1.3 dB group compared with the control group (**Figures 2C, D**). These results indicated that the cerebral activity of Bama pigs is more sensitive to underwater infrasound in the 8 Hz/145.4 ± 0.2 dB group and the 8 Hz/156.2 ± 1.0 dB group compared with the other four infrasound exposure groups.

### Increased incidence of subarachnoid hemorrhage after underwater infrasound exposure

**Figure 3:**
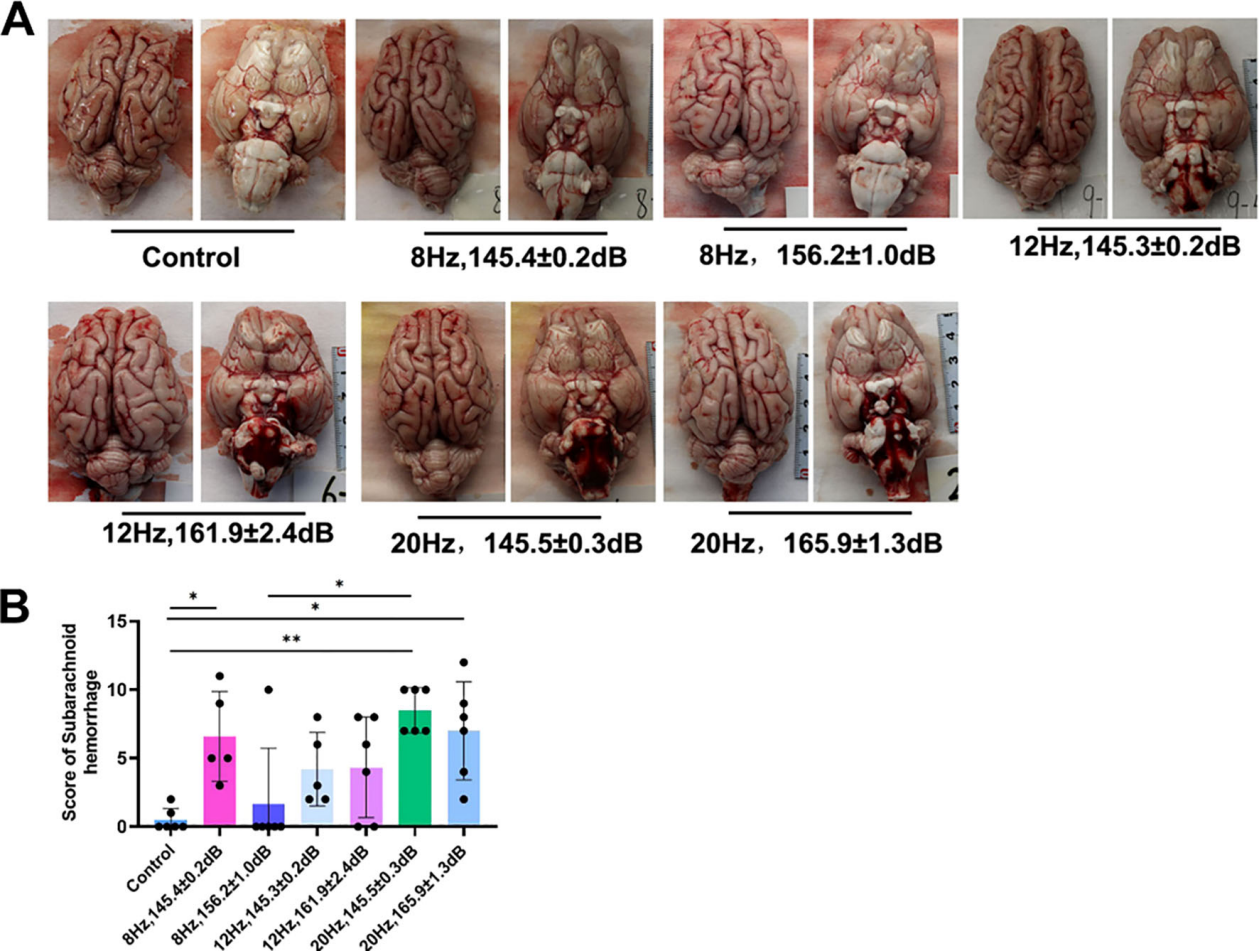
Evaluation of subarachnoid hemorrhages. **(A)** Gross analysis of subarachnoid hemorrhages. **(B)** Statistical analysis of subarachnoid hemorrhages. n = 6. Data represent means ± SD. *P <0.05, **P <0.01.

After the animals were sacrificed, gross anatomical examination was performed. Most notably, infrasound exposure could trigger subarachnoid hemorrhages of varying severity, as shown in **Figure 3A**. According to previous studies, subarachnoid hemorrhage scoring was performed (18DDI) (He et al., 2024, Lu et al., 2024, Sugawara et al., 2008). The results showed that, compared with the control group, the scores of all infrasound exposure groups were increased (**Figure 3B**). There were significant differences between the 20 Hz groups (145 ± 0.3 dB, 165.9 ± 1.3 dB) and the control group, and there was also a significant difference between the 20 Hz/145 ± 0.3 dB group and the 8 Hz/156.2 ± 1.0 dB group. Although the subarachnoid hemorrhage scores in other groups were higher than those in the control group, there was no significant difference. These results suggest that 20 Hz (145 ± 0.3 dB, 165.9 ± 1.3 dB) may significantly compromise the integrity of tight junctions within the cerebral vascular barrier and increase the risk of subarachnoid hemorrhage.

### Histological injury of brain after underwater infrasound exposure

**Figure 4:**
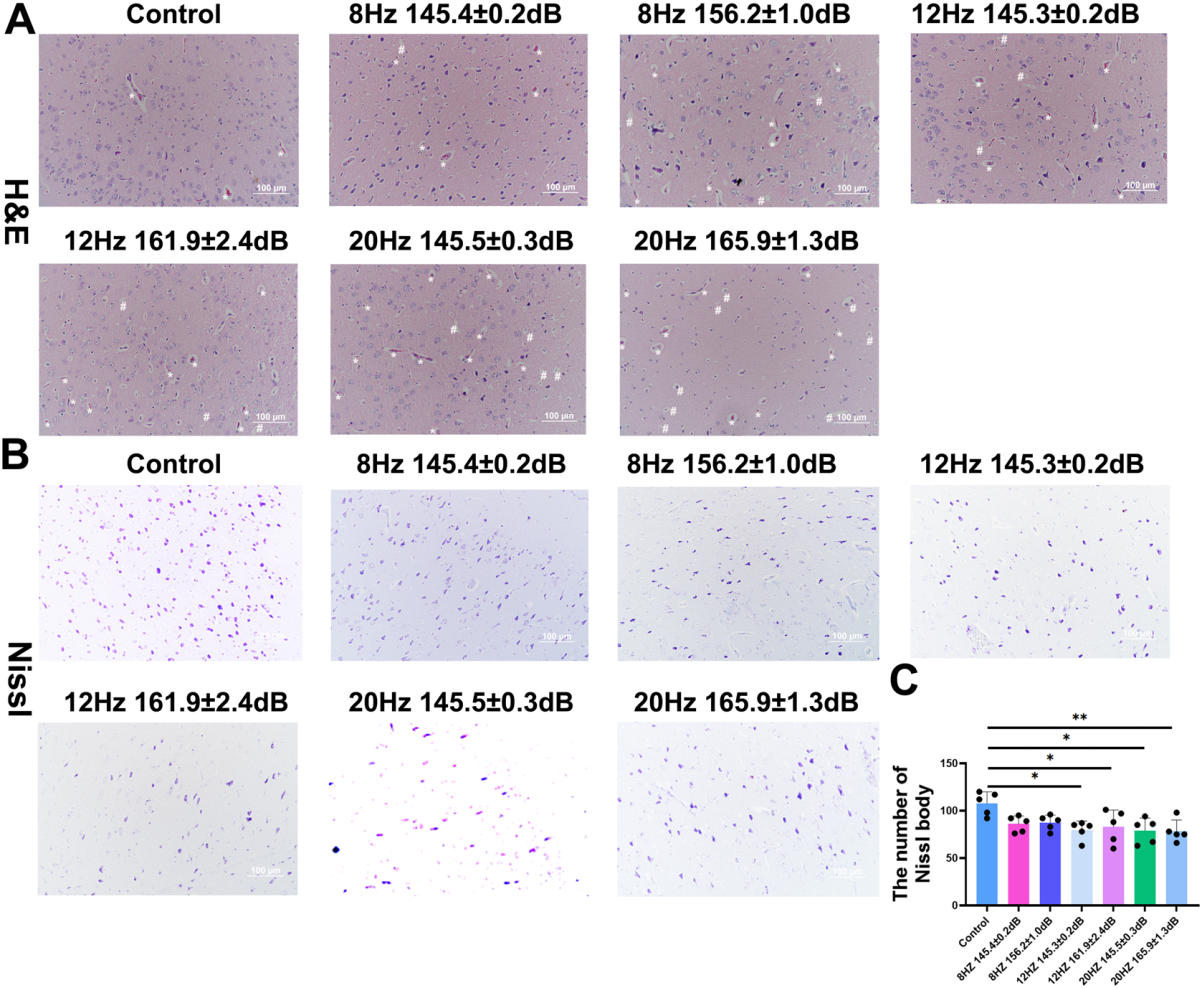
Morphological evaluation of underwater infrasound injury in the brain of Bama pigs. **(A)** H&E staining; **(B)** Nissl staining; **(C)** Statistical analysis of the density of Nissl bodies. The asterisks indicated red neurons with deeply stained cytoplasm; the hash marks indicated neuronal pyknosis and vacuolation. n = 6. Data represent means ± SD. *P <0.05, **P < 0.01.

To further evaluate the morphological injury induced by underwater infrasound, H&E staining and Nissl staining were performed. As shown in **Figure 4A**, H&E staining revealed that the morphological structure of the control group was intact and well-organized. In contrast, following underwater infrasound exposure, the cellular architecture was disrupted, accompanied by prominent red neurons with deeply stained cytoplasm, neuronal pyknosis, and vacuolation. Nissl staining provided additional direct evidence for assessing neuronal damage. The results showed that Nissl bodies were regularly and orderly distributed in the control group; however, after underwater infrasound exposure, the density of Nissl bodies per area was decreased (**Figures 4B, C**). Statistically significant differences were observed between the control group and the 12 Hz (145.3 ± 0.2 dB and 161.9 ± 2.4 dB) group, as well as the 20 Hz (145.5 ± 0.3 dB and 165.9 ± 1.3 dB) group, whereas no significant difference was found between the control group and the 8 Hz (145.4 ± 0.2 dB, 156.2 ± 1.0 dB) group. Collectively, these results demonstrate that with increasing frequency, underwater infrasound in the 12 Hz (145.3 ± 0.2 dB, 161.9 ± 2.4 dB) group and the 20 Hz (145.5 ± 0.3 dB, 165.9 ± 1.3 dB) group directly aggravated morphological damage to the brain and compromised neuronal survival.

### Reduced neuronal survival following underwater infrasound exposure

**Figure 5:**
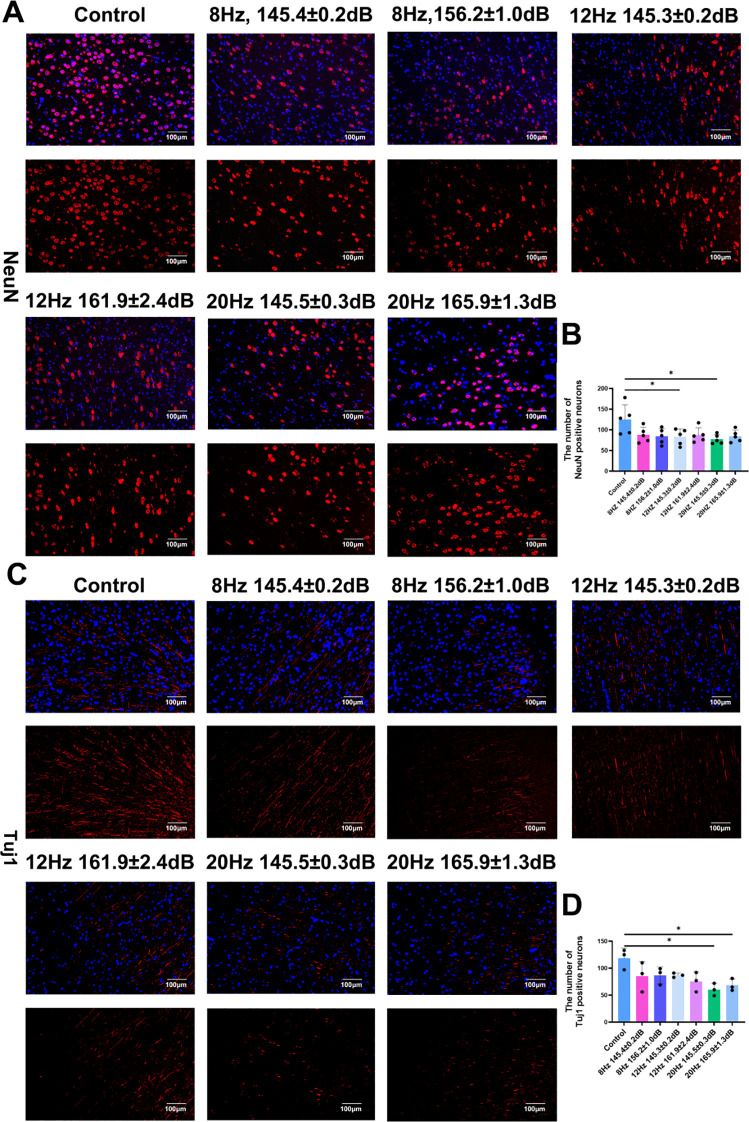
Neuronal injury evaluation of underwater infrasound exposure in the brain of Bama pigs. **(A)** Immunofluorescence staining of neurons in the injury brain. Blue, DAPI; red, NeuN; **(B)** Statistical analysis of NeuN^+^ neurons. n = 6; **(C)** Immunofluorescence staining of axons in the injury brain. Blue, DAPI; red, Tuj-1; **(D)** Statistical analysis of Tuj1^+^ neurons. n = 6. Data represent means ± SD. *P <0.05.

To further assess neuronal damage caused by underwater infrasound, the anti NeuN antibody was used to label neuronal nuclei. Consistent with the morphological injury, the number of NeuN-positive neurons in the underwater exposure groups was also decreased compared with the control group. After statistical analysis, there were significant differences between the control group and the 12 Hz/145.3 ± 0.2 dB, 20 Hz/145.5 ± 0.3 dB groups. However, there was no significant difference between the control group and the other underwater infrasound exposure groups (**Figures 5A, B**). In addition, the axon-specific marker Tuj-1 showed that the number of Tuj1 positive axons in the control group was significantly higher than in the underwater exposure groups, with significant differences between the control group and the 20 Hz/145.5 ± 0.3 dB group and the 20 Hz/165.9 ± 1. 3dB group (**Figures 5C, D**). Taken together, these findings reveal that underwater infrasound exposure in the 12 Hz/145.3 ± 0.2 dB, 20 Hz/145.5 ± 0.3 dB and 20 Hz/165.9 ± 1.3 dB groups could directly cause lead to neuronal damage.

### Increased apoptosis following underwater infrasound exposure

**Figure 6:**
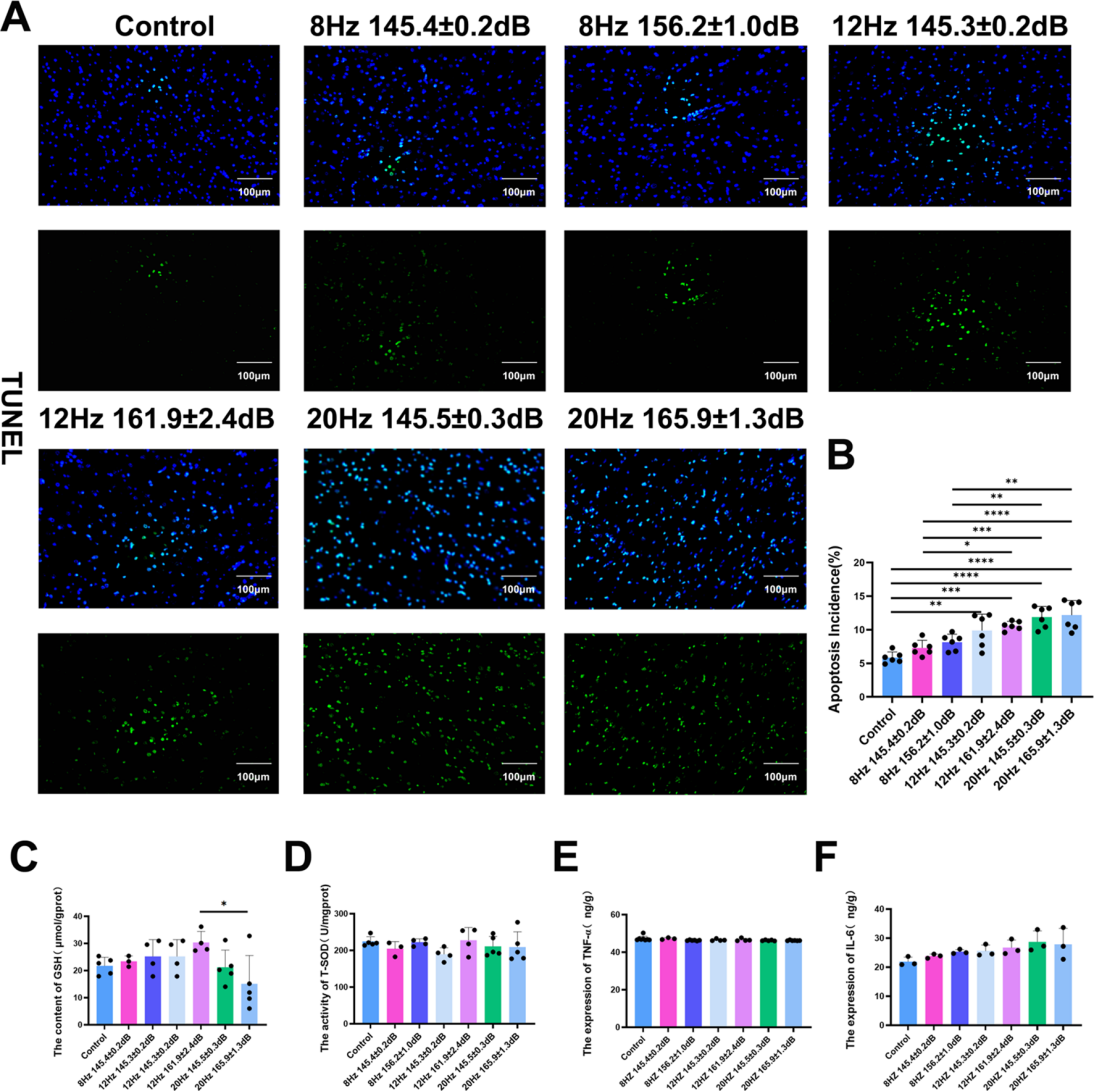
Evaluation of cell apoptosis and inflammation after being exposed to infrasound of underwater environment. **(A)** TUNEL staining of apoptotic cells; **(B)** Statistical analysis of TUNEL staining. n = 6; **(C)** Statistical analysis of the content of GSH. n = 6; **(D)** Statistical analysis of the activity of T-SOD. n = 6; **(E)** Statistical analysis of the expression of TNF-α. n = 6; **(F)** Statistical analysis of the expression of IL-6. n = 6. Data represent means ± SD. *P <0.05, **P < 0.01, ***P <0.001 and ****P <0.0001.

In previous study, it was reported that infrasound in an air environment could directly lead to cell apoptosis (Liu et al., 2012; Zhou et al., 2020). Then TUNEL staining was used to detect cell apoptosis after exposure to infrasound in an underwater environment. As shown in **Figures 6A, B**, the number of TUNEL-positive cells in the control group was relatively low. After underwater infrasound exposure, the number of TUNEL-positive cells increased, with a significant increase observed in the 12 Hz (145.3 ± 0.2 dB, 161.9 ± 2.4 dB) and 20 Hz (145.5 ± 0.3 dB, 165.9 ± 1.3 dB) groups compared to the control group, but no significant difference was observed between the 8 Hz (145.4 ± 0.2 dB, 156.2 ± 1.0 dB) groups and the control group. Thus, these results show that underwater infrasound in the 12 Hz (145.3 ± 0.2 dB, 161.9 ± 2.4 dB) and 20 Hz (145.5 ± 0.3 dB, 165.9 ± 1.3 dB) groups increased the number of TUNEL-positive apoptotic cells.

Furthermore, it was reported that infrasound in an air environment could activate oxidative stress responses and inflammatory reactions, which are closely associated with cell apoptosis (Pei et al., 2013). Therefore, we also measured the levels of major oxidative stress response indicators, including glutathione (GSH) and total superoxide dismutase (T-SOD), in the brain. The results showed that there were no significant changes in the levels of GSH and T-SOD between the control group and the underwater exposure groups (**Figures 6C, D**). Consistent with the results of oxidative stress responses, the major indicators of inflammatory reactions, TNF-α and IL-6, were also detected by ELISA, with no significant differences between the control group and the underwater infrasound exposure groups (**Figures 6E, F**). As shown by TUNEL staining, underwater infrasound exposure directly induced DNA fragmentation in the brain, a hallmark of apoptosis. This effect might be associated with the mechanical resonance of infrasound. Through mechanoreceptors on the cell surface, the mechanical vibration of infrasound could be directly converted into biological signals within the cell, further triggering cell apoptosis and neuronal injury. However, given the relatively short observation period, oxidative stress and subsequent inflammatory responses were not significant.

### Transcriptomic profiling of injured brain after underwater infrasound exposure

**Figure 7:**
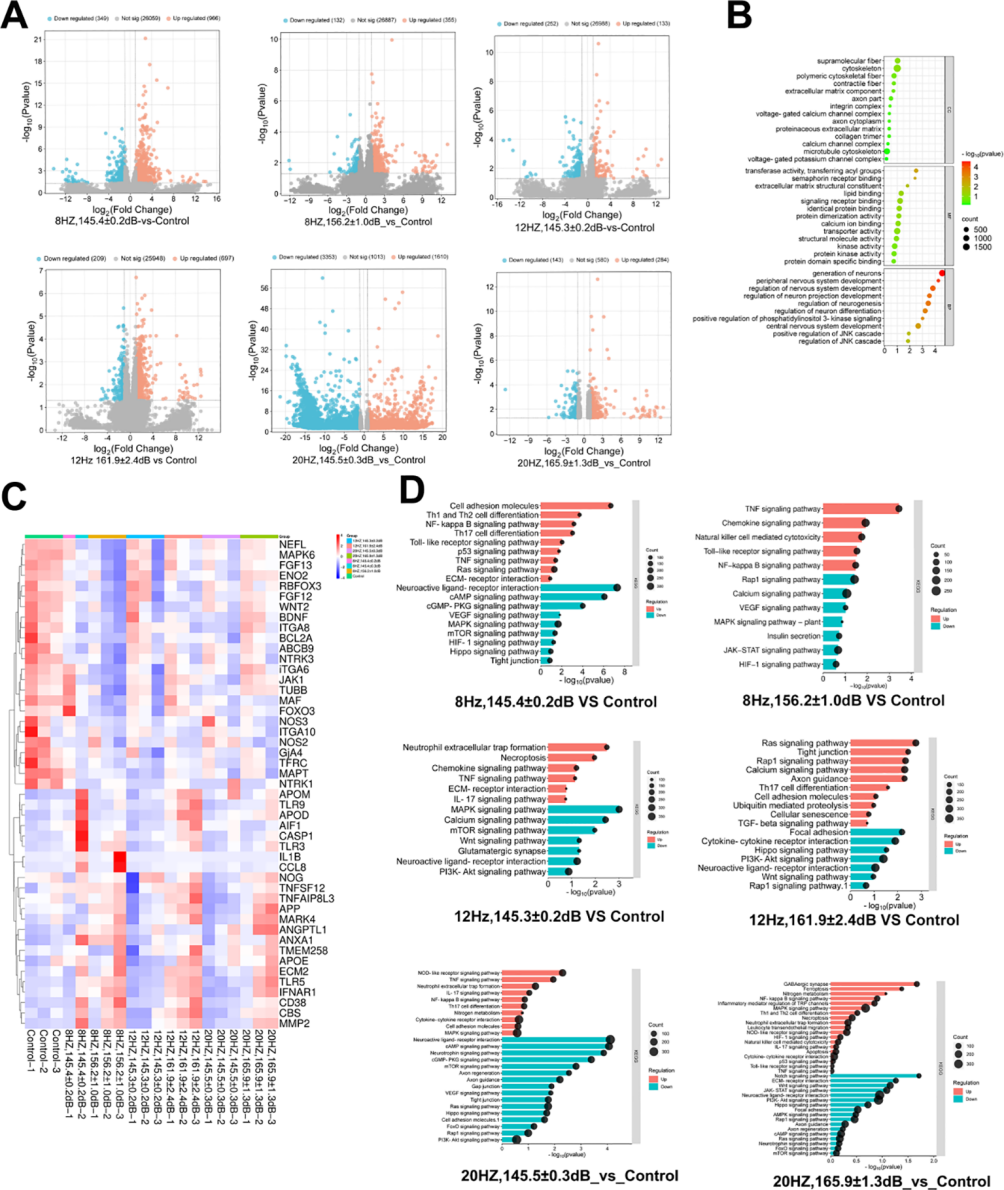
Transcriptome analysis to determine the mechanism of underwater infrasound in injured brain. **(A)** Expression of differentially expressed genes. **(B)** Gene Ontology (GO) analysis. **(C)** Significantly differentially expressed genes. **(D)** Kyoto Encyclopedia of Genes and Genomes (KEGG) analysis.

Finally, transcriptome analysis was used to explore the potential molecular mechanism of underwater infrasound. Differential expression gene (DEG) analysis showed that, compared with the control group, a series of genes were upregulated and downregulated in different underwater exposure groups (**Figure 7A**). To fully understand the biological processes, molecular functions, and cellular components associated with transcriptional changes in the porcine brain, we performed gene ontology (GO) enrichment analysis. GO enrichment analysis revealed significant involvement of cellular components (CC) associated with cytoskeletal organization and the extracellular matrix, molecular functions (MF) associated with protein binding and signaling, and biological processes (BP) associated with neurogenesis and neurodevelopment (**Figure 7B**).

Compared with the control group, apoptosis-related genes (*CD38*, *TNFSF12*, *TLR5*, *IFNAR1*) were upregulated in the 8 Hz/156.2 ± 1.0 dB, 12 Hz/161.9 ± 2.4 dB, and 20 Hz/165.9 ± 1.3 dB groups suggesting activation of antiviral and immune defense mechanisms. Vascular leakage-related genes (*MMP2, ANGPTL1*) was also upregulated in these same groups, which is associated with subarachnoid hemorrhage and its subsequent biological effects. In contrast, infrasound exposure caused downregulation of the anti-apoptotic gene *BCL2A*, suggested that acoustic stimulation may induce apoptosis in brain tissue. Downregulation of fibroblast growth factors (*FGF12*, *FGF13*), brain-derived neurotrophic factor (*BDNF*), and *WNT2* were also observed, which is involved in the potential regulation of neuronal development and regeneration pathways (**Figure 7C**).

KEGG pathway analysis was performed to identify signaling pathways affected by underwater infrasound. Compared with the control group, significant activation was observed in pathways related to TRP channels (which mediate inflammatory responses), ECM-receptor interaction, IL-17 signaling, and TGF-a signaling, particularly in the high-intensity exposure groups. These pathways are involved in inflammatory responses, extracellular matrix remodeling, and immune regulation within brain tissue, suggesting potential alterations in neuroinflammation, tissue repair, and neurodevelopment. Collectively, these results indicate that acoustic stimulation exerts significant effects on the pig brain, primarily manifested as immune responses, inflammation, cell survival, tissue remodeling, neurodevelopment, and hypoxic stress, with the magnitude of these effects closely associated with acoustic simulation intensity. These findings provide a critical molecular foundation for further investigations into the impact of acoustic stimulation on brain function.

## Discussion

Infrasound is a type of low-frequency acoustic noise and has become a new public health hazard (Baliatsas et al., 2016). Accumulating evidence has demonstrated that infrasound can cause dysfunction in multiple organs, especially the CNS (Ascone et al., 2021; Zhang et al., 2023). It has been shown to directly impair learning and memory (Wang et al., 2015). This knowledge of infrasound in organisms was mainly derived from studies conducted in air environments. However, the bioeffects of underwater infrasound may differ from those in air. Therefore, a comprehensive understanding of the effects of underwater infrasound on the cerebral function of humans or large animals is still lacking (Laurer et al., 2002).

The infrasonic transducer used in the present study was placed at a depth of 10 m underwater and emitted a continuous 20 Hz wave. Meanwhile, two hydrophones were used for SPL calibration. Hydrophone 1 was placed vertically aligned with the infrasonic transducer to measure the sound pressure level emitted by the transducer; Hydrophone 2 was placed horizontally aligned with the Bama pig to measure the sound pressure level received by the animal. As the animals and the infrasonic transducer were positioned on the same horizontal plane, the sound field around the animals was not influenced by the standing wave ratio. After theoretical calculations, both the animals and the two hydrophones were in far-field acoustic conditions, and harmonic distortion was less than 2%. In this condition, at the depth of 10 m underwater, a sound pressure level of at least approximately 226 dB re 1 μPa was required to generate cavitation. In our experiment, the sound pressure level did not exceed 170 dB; therefore, cavitation could not occur.

Firstly, cerebral activity was assessed using EEG after infrasound exposure. As shown in **Figure 2**, underwater infrasound exposure directly altered EEG waveforms, including abundant paroxysmal abnormal discharges. Further statistical analysis revealed that only the 8 Hz/145.4 ± 0.2 dB group and the 8 Hz/156.2 ± 1.0 dB group caused significant alterations mainly in the standardized energy values of α- and θ-waves in the central and frontal regions, whereas no significant histological injury was observed under these conditions. This may be explained by the fact that, during moderate anesthesia or sedative-hypnotic states, θ waves typically occur within the 5 Hz–7 Hz range. Under these conditions, exposure to 8 Hz infrasound may directly interfere with the animal’s endogenous θ brain activity, potentially inducing standardized energy values of θ-waves in both the central and frontal regions. In addition, α waves, with a frequency of 8 Hz–13 Hz, are close to 8 Hz infrasound and were also affected in the frontal region. Functionally, the frontal region is primarily involved in higher cognitive functions, including motor planning, executive control, attention, working memory, and language expression, while the central region serves as the core hub for sensorimotor integration, coordinating voluntary movement with sensory feedback. Therefore, these results are consistent with previous studies showing that infrasound leads to dysfunction of the human brain, including nervousness, fearfulness, disorientation, and learning and memory deficits.

Histological and pathological evaluation further demonstrated that underwater infrasound could directly cause pathological damage to the brain. As shown in **Figure 3**, compared with the control group, infrasound stimulation led to irregular cellular arrangement in the brain and directly decreased the number of neurons and Nissl bodies in the 12 Hz and 20 Hz groups. These results were further confirmed by immunostaining of neuronal markers (NeuN), axonal markers (Tuj-1), and TUNEL staining (**Figures 4**-**6**). The results showed that, compared with the control group, 12 Hz and 20 Hz underwater infrasound caused a decrease in the number of neurons and an increase in cell apoptosis. However, no significant difference was observed in histological injury between the 8 Hz groups and the control group. We analyzed the underlying reasons: the biological mechanism of infrasound may involve bioresonance. In a previous study, Laksari and colleagues used *in vivo* MRI data and a lumped-parameter model to simplify the brain as a rigid body mass and the meninges, blood vessels, and other connective tissues as springs and dampers. By applying the classical second-order “mass–spring–damper” system equation, they calculated the overall resonance frequency of the human brain to be approximately 15 Hz (Laksari et al., 2015). In the present study, the infrasound frequencies of 12 Hz and 20 Hz were closer to 15 Hz, which may explain why 12 Hz and 20 Hz infrasound at higher sound pressure levels led to more pronounced histological injury compared with 8 Hz.

Furthermore, it has been reported that infrasound exposure under air conditions can also activate neuroinflammation and oxidative stress. However, in the injured brain, no significant differences were observed in the levels of the proinflammatory cytokines TNF-α and IL-6, or in the oxidative indicators GSH and T-SOD (**Figures 6C–F**). This may be due to the fact that the samples were collected approximately 2 h after infrasound exposure. In general, indicators of oxidative stress and proinflammatory cytokines typically appear several hours to days after injury. Therefore, prolonged observation will be conducted to evaluate long-term biological effects.

In addition, the potential molecular mechanism of cerebral injury induced by underwater infrasound was explored using transcriptome sequencing. Compared with the control group, key signaling pathways and genes associated with necroptosis, inflammatory responses, and cell apoptosis were significantly upregulated. In contrast, key signaling pathways and genes associated with anti-apoptosis, neuroprotection, and regeneration were significantly downregulated (**Figure 7**). Specifically, the vascular leakage-related genes *MMP2* and *ANGPTL1* were upregulated in the 8 Hz/156.2 ± 1.0 dB, 12 Hz/161.9 ± 2.4 dB, and 20 Hz/165.9 ± 1.3 dB groups, which are directly associated with subarachnoid hemorrhage and its subsequent biological effects. Meanwhile, the anti-apoptotic gene-*BCL2A* and the neuroprotective genes *FGF12*, *FGF13*, BDNF, and *WNT2* were significantly decreased. These results are consistent with the histological evaluations and provide a basis for further mechanistic studies of underwater infrasound.

Finally, we focused on the immediate injury caused by underwater infrasound. However, some blood and histological indicators, such as oxidative stress and inflammatory responses, appear relatively late. This is one of the limitations of this study; additionally, it has been reported that infrasound may cause adverse effects such as vertigo and impaired attention. However, due to limitations in the experimental site conditions, we were unable to perform vestibular function or other neurofunctional assessments on the animals. This was another limitation of this study. In the future, we will prolong the observation time and expand the quantifiable functional evaluation system to systematically assess the comprehensive biological effects of underwater infrasound on the organism.

In conclusion, cerebral injury caused by underwater infrasound was systematically investigated. The potential biological effects included two aspects. First, infrasound could directly damage the endothelial barrier of surface blood vessels in brain tissue, thereby leading to subarachnoid hemorrhage. Second, infrasound could act on mechanoreceptors on the cell membrane surface, convert signals into intracellular signals, and directly induce cell apoptosis. Compared with the control group, underwater infrasound exposure led to altered EEG waveforms, induced histological injury, and increased cell apoptosis. This process activated cell apoptosis pathways and key genes while suppressing those involved in neuroprotection and regeneration. In the future, the underlying mechanisms and long-term effects of underwater infrasound exposure on the brain will be further investigated.

## Data Availability

The original contributions presented in the study are publicly available. This data can be found here: https://www.jianguoyun.com/p/Da6qQdoQ66SdDhilgqkGIAA and https://www.jianguoyun.com/p/DR-8VwMQm6edDhirgqkGIAA.

## Supplementary material

Supplementary Figure 1 The physical layout and EEG recording electrodes placement. **(A, B)** The physical layout diagram of diving meter, hydrophone 2; **(C)** The placement of EEG recording electrodes.

Supplementary Figure 2 Zoomed-in EEG waveforms after various infrasound exposure. Arrows denoted the abnormal discharges on EEG.
