# Supplementary figures and images for: Systematic evaluation of cerebral injury stimulating by underwater infrasound
Source: Front Physiol. 2026 May 13;17:1803881. doi: 10.3389/fphys.2026.1803881 (PMC13212229; doi:10.3389/fphys.2026.1803881)

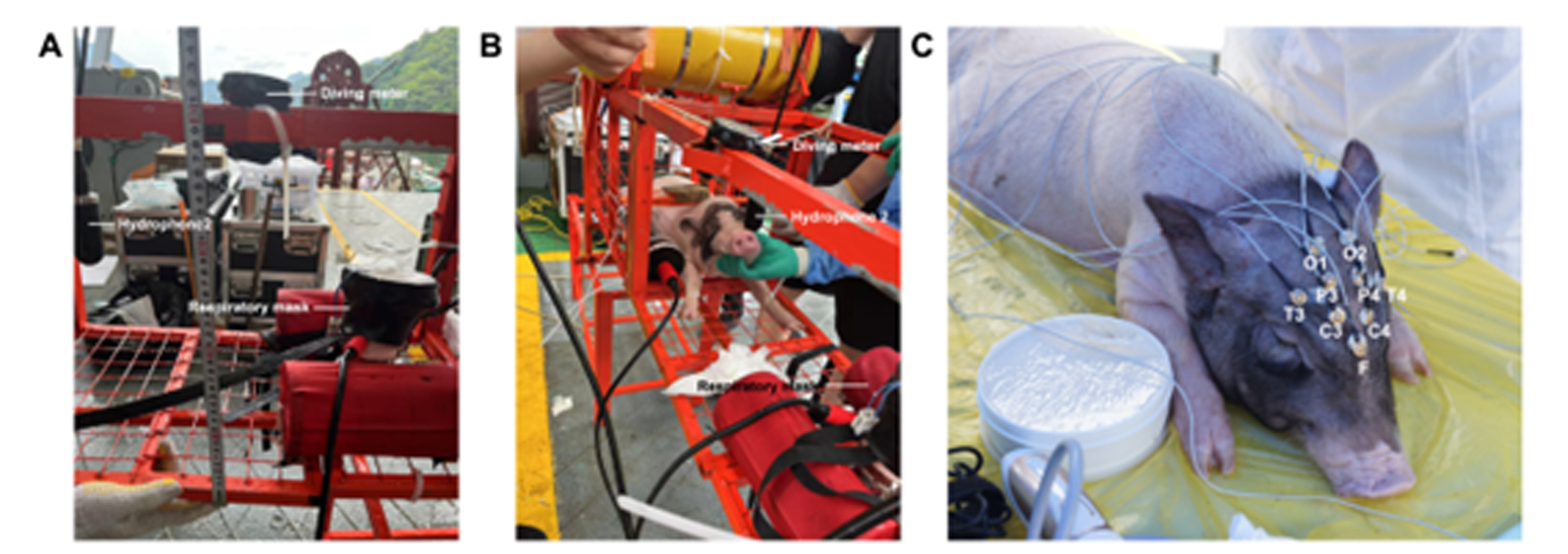

Supplement: Supplementary Figure 1 — The physical layout and EEG recording electrodes placement. (A, B) The physical layout diagram of diving meter, hydrophone 2; (C) The placement of EEG recording electrodes. [file Image1.tif]

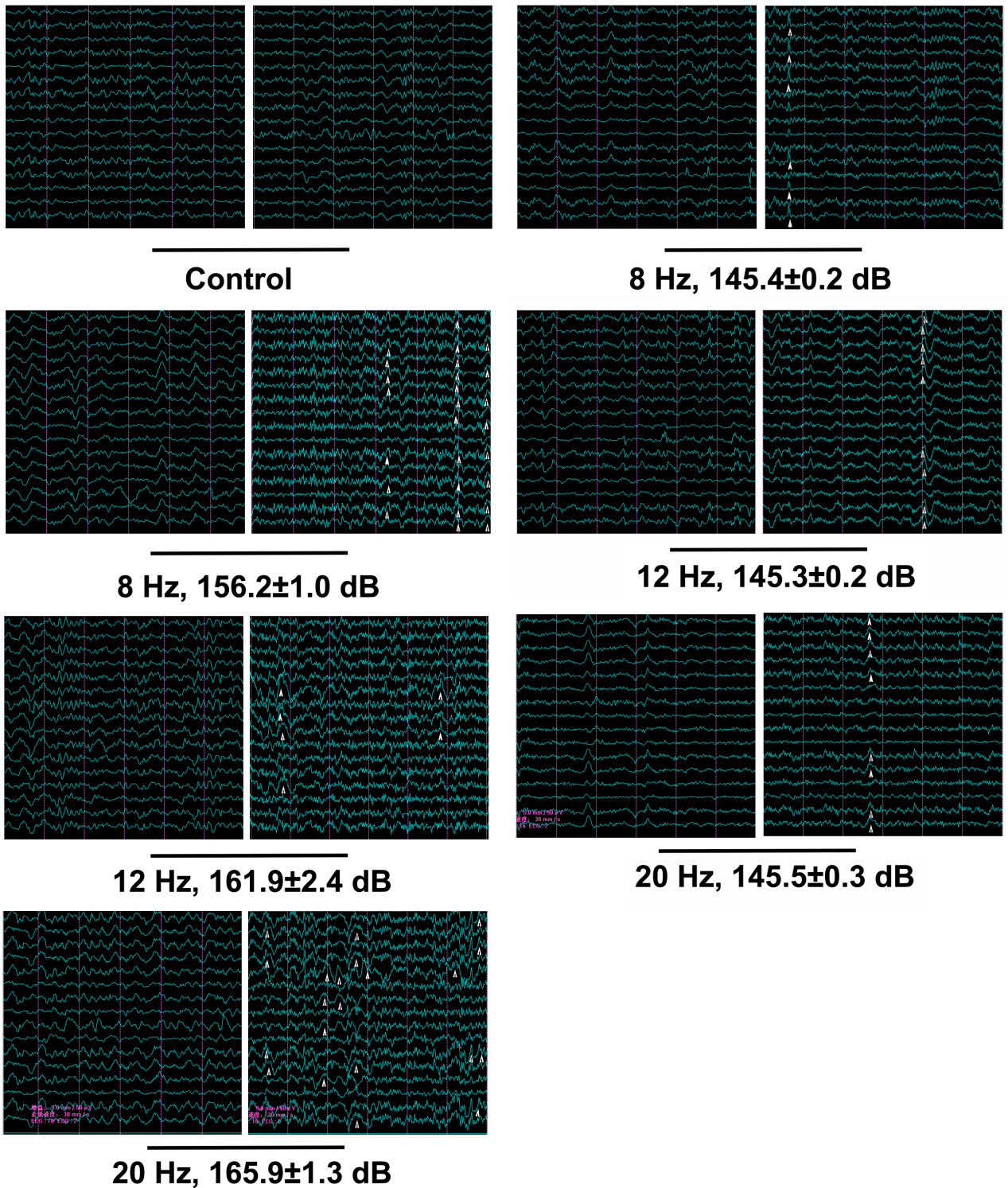

Supplement: Supplementary Figure 2 — Zoomed-in EEG waveforms after various infrasound exposure. Arrows denoted the abnormal discharges on EEG. [file Image2.tif]
